# Beta Oscillatory Changes and Retention of Motor Skills during Practice in Healthy Subjects and in Patients with Parkinson's Disease

**DOI:** 10.3389/fnhum.2017.00104

**Published:** 2017-03-07

**Authors:** Aaron B. Nelson, Clara Moisello, Jing Lin, Priya Panday, Serena Ricci, Andrea Canessa, Alessandro Di Rocco, Angelo Quartarone, Giuseppe Frazzitta, Ioannis U. Isaias, Giulio Tononi, Chiara Cirelli, M. Felice Ghilardi

**Affiliations:** ^1^Department of Physiology, Pharmacology and Neuroscience, City University of New York School of MedicineNew York, NY, USA; ^2^Department of Informatics, Bioengineering, Robotics and System Engineering, University of GenoaGenoa, Italy; ^3^Fondazione Europea di Ricerca BiomedicaCernusco sul Naviglio, Milan, Italy; ^4^The Fresco Institute at New York University School of MedicineNew York, NY, USA; ^5^Centro Neurolesi, University of MessinaMessina, Italy; ^6^Ospedali Gravedona e RiunitiGravedona, Italy; ^7^Department of Neurology, University Hospital and Julius-Maximillian-UniversityWuerzburg, Germany; ^8^Parkinson Institute ASST Gaetano Pini-CTOMilan, Italy; ^9^Department of Psychiatry, University of Wisconsin-MadisonMadison, WI, USA

**Keywords:** plasticity, reaching movements, ERD, ERS

## Abstract

Recently we found that modulation depth of beta power during movement increases with practice over sensory-motor areas in normal subjects but not in patients with Parkinson's disease (PD). As such changes might reflect use-dependent modifications, we concluded that reduction of beta enhancement in PD represents saturation of cortical plasticity. A few questions remained open: What is the relation between these EEG changes and retention of motor skills? Would a second task exposure restore beta modulation enhancement in PD? Do practice-induced increases of beta modulation occur within each block? We thus recorded EEG in patients with PD and age-matched controls in two consecutive days during a 40-min reaching task divided in fifteen blocks of 56 movements each. The results confirmed that, with practice, beta modulation depth over the contralateral sensory-motor area significantly increased across blocks in controls but not in PD, while performance improved in both groups without significant correlations between behavioral and EEG data. The same changes were seen the following day in both groups. Also, beta modulation increased within each block with similar values in both groups and such increases were partially transferred to the successive block in controls, but not in PD. Retention of performance improvement was present in the controls but not in the patients and correlated with the increase in day 1 modulation depth. Therefore, the lack of practice-related increase beta modulation in PD is likely due to deficient potentiation mechanisms that permit between-block saving of beta power enhancement and trigger mechanisms of memory formation.

## Introduction

Movement is accompanied by EEG changes of beta oscillations over the sensory-motor areas, with a power decrease before movement onset, a negative peak during execution (event-related *desynchronization*, ERD) and a post-movement increase (*event-related synchronization*, ERS) (Pfurtscheller and Lopes da Silva, 1999; Toma et al., 2002). These changes occur for a variety of movement tasks, including a reaching movement task in a choice reaction time paradigm (Tombini et al., 2009; Perfetti et al., 2011; Moisello et al., 2015b). In a recent study, we have found that the amplitude of these oscillations significantly increases during a 40-min practice in a task with movements to unpredictable targets with negligible learning requirements (Moisello et al., 2015b). The increase, which was most evident in the ERS component, was not due to the increase of mean power, as we also found an increase of the modulation depth of the ERD-ERS peak-to-peak amplitude, a measure that is independent from changes of mean power. Finally, the changes in movement-related beta modulation did not correlate with the increased speed or improvements in other kinematic measures that occurred during the task.

To speculate on the origin of this practice-dependent increase, two lines of evidence must be taken into account. On one hand, beta modulation likely reflects the interplay of sensory and motor regions' activities during motor performance (Shimazu et al., 1999; Cassim et al., 2000) with activation of motor areas and attenuation of sensory afferents during movement followed by re-activation of somatosensory areas and idle state of the motor areas. On the other side, increases of beta power have been associated with high GABA levels in animal and human studies (Jensen et al., 2005; Roopun et al., 2006; Yamawaki et al., 2008; Hall et al., 2010, 2011; Muthukumaraswamy et al., 2013; Rossiter et al., 2014) and decreases in cortical excitability in humans (Hsu et al., 2011; Noh et al., 2012; McAllister et al., 2013). Indeed, these two lines of evidence are not incompatible: the repetitive pattern of alternate activation and inactivation of the sensory and motor areas during the continuous, uninterrupted practice of a task likely triggers the induction of long term potentiation (LTP) that may reinforce existing sensory-motor memories (or internal models) or create new ones, finally resulting in skill enhancement. Thus, the increase of movement-related beta modulation during the task could represent the progressive saturation of the mechanisms related to LTP-like plasticity. This interpretation is supported by recent observation that the beta modulation amplitude is linked to movement adaptation to new sensory-motor transformations and thus to formation of new internal models (Tan et al., 2016). Other support comes from our previous finding that movement-related beta modulation does not significantly increase with practice in patients with Parkinson's disease (PD) (Moisello et al., 2015b), a disease that is accompanied by a decrease of skill retention (Marinelli et al., 2009; Bedard and Sanes, 2011; Isaias et al., 2011; Moisello et al., 2015a) and by deficits in the induction of use-dependent and LTP-like plasticity (Morgante et al., 2006; Kishore et al., 2012; Koch, 2013). Therefore, if the increase of movement-related beta modulation during the task parallels the early induction of LTP, one should expect increases of beta modulation within a set of consecutive movements but a partial decrease in the intervals between successive sets. Because of their deficits in plasticity and retention, we expect that patients with PD should show either a lack of this within-set increase of beta modulation or, alternatively, a faster between-set decrease. Furthermore, on a second exposure to the task the following day, enhancement of performance should occur in normal subjects but not in patients with PD, while beta modulation should show similar changes in both groups as in day 1.

Here we test these hypotheses and record EEG in patients with PD and age-matched controls during two sessions of 40-min reaching task divided in fifteen blocks of 56 movements each performed on two consecutive days. We focused our analyses on beta modulation depth, a measure independent from changes of mean power, and on the activity of the electrodes over the left sensory-motor area where practice-related changes are more notable (Moisello et al., 2015b).

## Methods

### Subjects

Eleven patients with PD (one woman, age: mean 59.1 ±SD 5.8 years, Hoehn & Yahr stage: 2.0 ± 0.2; disease duration: 5.0 ± 2.1 years; Unified Parkinson's Disease Rating Scale (UPDRS)–III (motor section) score: 20.9 ± 8.5; Levodopa Equivalent Daily Dose: 582.5 ± 221.2) and 13 age-matched controls with normal neurological examination (six women, age: 57.5 ± 8.2 years) participated in this study. All subjects were right-handed as determined by the Edinburgh inventory (Oldfield, 1971) and had normal or corrected vision. Controls had no history of neurological or psychiatric disorders. Patients were tested in ON state, in their best condition, approximately 1 h from their morning dose of dopaminergic medications. All patients successfully completed the entire protocol (see below) without experiencing fatigue. The experiments were conducted with the approval of our Institutional Review Board. Written informed consent was obtained from all participants.

### Experimental design

All subjects performed the identical session in two consecutive days, both sessions starting around 9 am. At the beginning of each session, subjects were outfitted a 256-channel EEG cap (Electrical Geodesics Inc.). Three minutes of resting state EEG were recorded before (PRE) and after (POST) performance of the motor task. During the resting state, subjects were asked to relax, to keep their eyes open and to fixate a black circle in the center of a computer screen. The methods and results of the analyses of the resting state are reported in the Supplemental Material.

### Motor task

**Figure 1:**
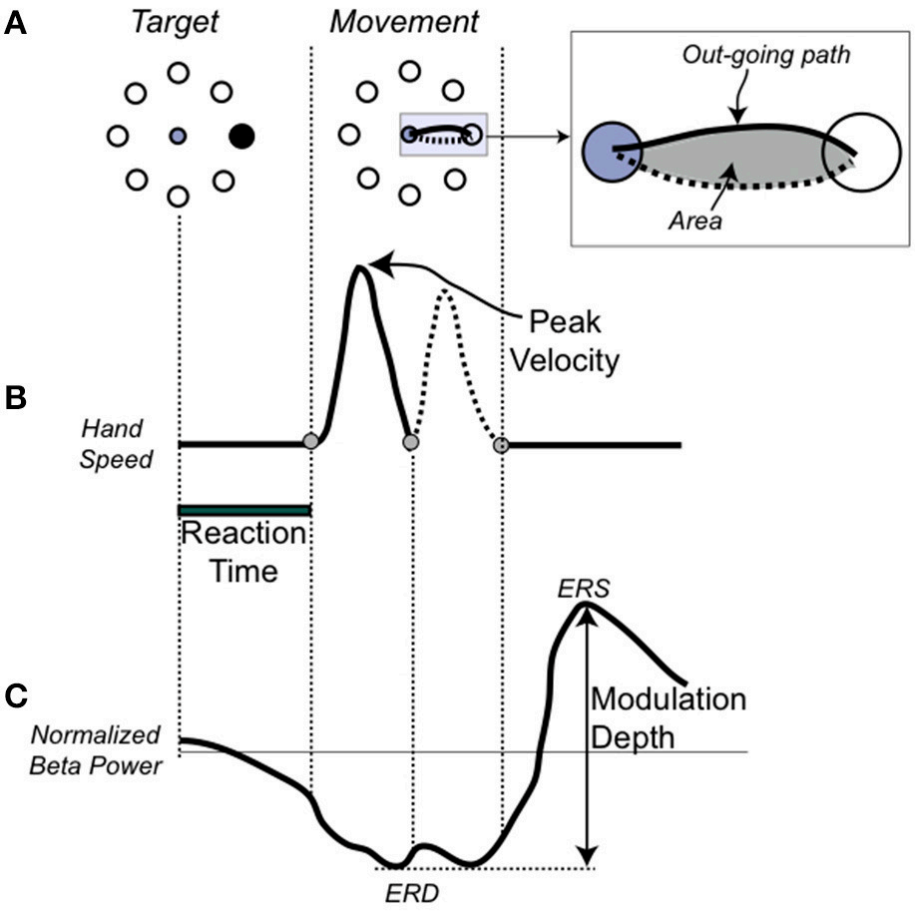
**Single movement, performance, and EEG measures (A)**. On the Left, the target array and in black, the target to be reached. In the center, the movement trajectory to the target. In the inset, the depiction of the hand path area (in gray) with the outgoing portion in a solid line and the return portion in dotted line. **(B)** Temporal profile of the trajectory. **(C)** Schematic representation of the beta modulation during a single movement with the definition of the event-related desynchronization (ERS), as the minimum amplitude of the beta power, event-related synchronization (ERD), as the maximum amplitude of the beta power, and modulation depth.

General features of the motor task have been reported in previous studies (Ghilardi et al., 2000b, 2009; Perfetti et al., 2011; Moisello et al., 2015b). Briefly, subjects moved a cursor on a digitizing tablet (sampling rate 200 Hz) with their right hand to targets presented on a screen. Targets were eight circles (1 cm radius) equidistant (4 cm) from the central starting point in the center of the screen (Figure 1A). The eight target circles and the position of the cursor on the screen were visible at all time. Upon presentation, one of the targets turned black for 400 ms. Targets blackened in random order, at 1.5 s intervals. Instructions were to move as soon as possible, to make overlapping out-and-back movements from the starting point to the presented target without corrections, as fast and accurately as possible, and to reverse sharply within the target without stopping. Subjects were also asked to move as soon as possible, thus minimizing reaction time, but also to avoid anticipation or guessing. Subjects performed a total of 840 movements in 15 blocks of 56 movements each. After each block, subjects paused for an interval varying from 13 to 249 s. Each session lasted approximately 40 min. Before the first testing session, all subjects were trained to reach a hit rate of 95%. This was usually accomplished in <10 min.

As in previous publications (Ghilardi et al., 2000a, 2003; Perfetti et al., 2011), we analyze each trajectory and computed several spatial and temporal measures for each movement. Here we focused on and report results for: reaction time (time from target appearance to movement onset), amplitude of peak velocity, normalized hand-path area (the area enclosed by the hand path divided by the squared path length) (Huber et al., 2006; Moisello et al., 2008) (Figure 1B). These three measures were selected because they reflect improvement in following different parts of the instructions. Decreases in normalized hand path represent more overlap of the out and back movement segments, a measure of spatial accuracy and inter-joint coordination; decreases in reaction time is about moving sooner after the target; increases in the peak velocity reflect an increased role of the feed-forward commands and a decrease of feed-back mechanisms in movement production.

### EEG recording

EEG was recorded for the entire duration of the two experimental sessions. Data were collected at a sampling rate of 1000 Hz using the high impedance amplifier Net Amp 300 and Net Station 4.3 (Electrical Geodesics Inc.). Impedances were kept below 50 kΩ. From the original 256 electrodes, 73 located on the cheeks and on the neck were removed and the recordings from the remaining 183 electrodes were used for analysis. During the recording, EEG signal was referenced to Cz electrode. For analysis, data were down-sampled to 250 Hz and re-referenced to the average across the 183 electrodes.

### Preprocessing

We preprocessed the data with NetStation 4.3 software (Net Station EEG Software, RRID:nlx_155825, Electrical Geodesics Inc.) and the Matlab-based public license toolboxes EEGLAB (RRID:nif-0000-00076, Delorme and Makeig, 2004). Subsequent analyses also included functions from the Fieldtrip toolbox (RRID:nlx_143928, Oostenveld et al., 2011). Briefly, the continuous EEG signal was filtered between 0.5 and 80 Hz, with a notch filter at 60 Hz. Channels affected by bad scalp-electrode contact were visually identified and replaced with spherical spline interpolation (number of bad channels, mean ±SD, Controls: 1.9 ± 1.8; PD: 1.3 ± 1.3; *p* > 0.1). EEG recorded during the motor performance was segmented into 3-s epochs aligned with movement onset (−1 to +2 s). Epochs containing sporadic artifacts (abnormal tension bursts, cough or similar) were rejected by visual inspection. Stereotypical artifacts, such as blinks, eye movements and muscle tension, were removed by Independent Component Analysis (Makeig et al., 2004; Onton and Makeig, 2006).

### EEG during the motor task

#### EEG analysis

**Figure 2:**
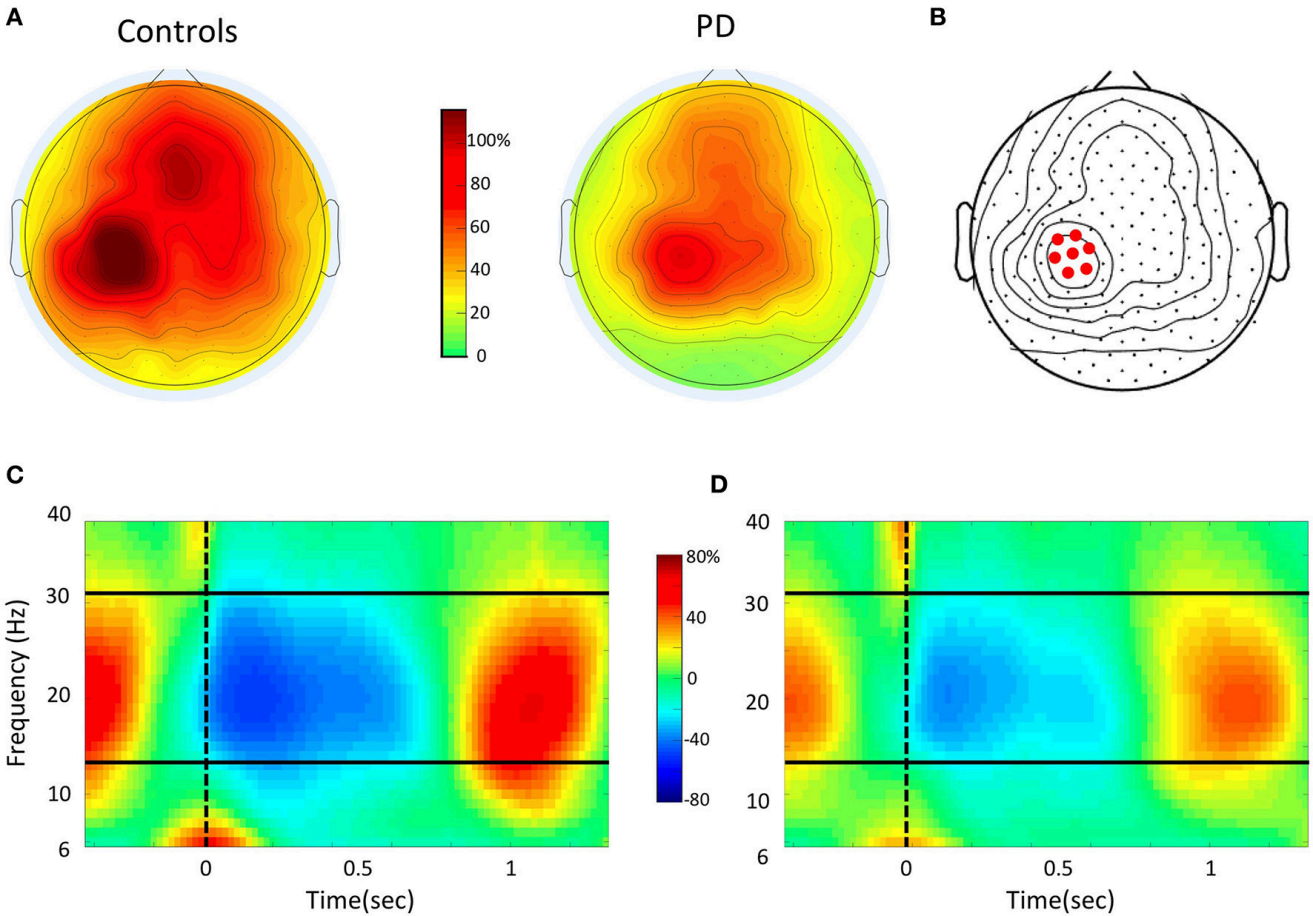
**EEG data analysis (A)**. Topographic distribution of the mean beta power (15–30 Hz) modulation depth (% change), as measured from maximal event-related desynchronization (ERD) to maximal event-related synchronization (ERS) in the Controls and PD groups. Topographies are averaged over all trials in each subject. The overall modulation is smaller in PD. However, in both maps there is a local maximum in Left sensory-motor area. **(B)** Identification of the left sensory-motor region of interest (ROI). The electrode with maximal modulation in the ROI is shown together with the six immediate neighbor electrodes. **(C,D)** Time-frequency plot for the event-related spectral change over the Left ROI obtained by averaging all trials of the control **(C)** and PD **(D)** groups. On the X-axis, 0 and the vertical dotted line indicate the time of the movement onset. The solid horizontal lines indicate the limits of the beta range. Notice that the strongest power modulation occurs in the beta range.

After preprocessing, all artifact-free trials from the motor task were submitted to time-frequency and statistical analyses. For all channels, we computed time-frequency representations in the range from 6 to 40 Hz using a short-time Fourier transform approach (Hanning taper, time step-size of 20 ms, 5 cycles adaptive window width, 1 Hz frequency step). For this study, we focused on beta oscillations, i.e., the range from 15 to 30 Hz. Indeed, this is the rhythm that undergoes the strongest and most consistent modulation during movement in the sensory-motor regions, as shown previously (Kilavik et al., 2013; Tan et al., 2014; te Woerd et al., 2014; Moisello et al., 2015b) and also in the present data (Figure 2D). As the movement-free time interval between consecutive movements is rather short, change in oscillatory power during movement was defined as percent change with respect to an average power value computed over the two entire motor sessions. As previously reported in numerous studies, beta power starts decreasing before movement onset, reaches a negative peak (ERD) during movement execution and finally shows a characteristic rebound (ERS) after the movement end (Figure 1C). To identify the sensors showing the strongest beta modulation depth, we averaged the normalized beta band power for all valid trials and plotted the scalp distribution of the difference between maximal ERD and ERS in each group (Figures 2A,B). As previously reported, in both groups, significant beta modulation depth was found in three areas, involving left and right parietal electrodes as well as medial frontal electrodes (Moisello et al., 2015b) (Figures 2A,B). Here we focused the analyses on the Left parietal area where practice-related changes are more notable (Moisello et al., 2015b). Briefly, we identified the electrode with the maximum beta modulation over the Left parietal area and we and included the six immediate neighbor electrodes to define the region of interest (ROI) (Figure 2C). The averaged beta power values over the seven electrodes for each block were used to define the time course of modulation depth, computed as the difference between positive and negative peak, across trials and blocks. Specifically, modulation depth was computed after averaging the power across each block as well as over the first 16 trials (early trials) and the last 16 trials (late trials) of each block trials (see also statistical analysis and results).

#### Data and statistical analyses

For each subject, we discarded from both kinematic and EEG analyses the movements that did not meet one of the following criteria: movement or reaction time within 2 SD of each subject's mean; movements with directional error >22°; previous movement ending 100 ms or less from the current target presentation. First, we compared data of day 1 and day 2; therefore, behavioral indices and modulation depth values were averaged across blocks and then mixed model, repeated-measure ANOVAs were performed with Group (PD, controls), Blocks (15) and Day (1 and 2) as main effects. We then ascertain whether there were differences between the 16 trials at the beginning (early trials) and the 16 at the end of each block (late trials). As beta modulation was similar in day 1 and 2, for this analysis we combined the data from the 2 days to increase the number of trials and the signal-to-noise ratio. This procedure allowed us to average a maximum of 32 trials for the early and the late trials of each block. After trial rejection, the average number of trials used was 24.9 (±0.22) for the controls and 25.1 (±0.32) for the patient group. We performed repeated-measure ANOVA on beta modulation depth values with trials (early, late) and block as repeated measures for the two group separately and then we then performed mixed model ANOVAs with trials and blocks as repeated measure and groups as main effects. We also performed repeated-measure ANOVAs on delta (difference in modulation depth between the early and late trials of each block), savings (difference between early trials of block 1 and those of all the other blocks) and between-block reset (modulation difference between the late trials of a block and the early trials of the next one) with blocks and group as main effects. Significant effects and interactions (alpha <0.05) were explored further using Bonferroni corrected *post-hoc* tests.

### Resting state EEG

#### EEG analysis and statistics

Analyses were restricted to the seven electrodes that we used to define the time course of modulation depth over the Left sensory-motor region. For the sake of consistency, we used the same analytical approach described for the movement-related EEG changes to extract time-frequency representations for each clean resting state data epoch in the ROI. The resulting data were then averaged over each time point and each epoch, to obtain an average beta power value for four time points, two before (PRE1) and after (POST1) the day 1 session and the other two before (PRE2) and after (POST2) the day 2 session. To verify whether significant changes had occurred, for each group we used a repeated measure ANOVA on the power values, with two time levels: day (1 and 2) and practice (PRE and POST). Significant effects and interactions were further explored with Bonferroni-corrected *post-hoc* tests (alpha = 0.05). Pearson product correlations were computed between EEG-derived and other measures.

## Results

### Performance changes across days

All participants completed the two 40-min sessions without reporting any difficulty or fatigue. In general, as in previous publications (Moisello et al., 2015b), movements were mostly straight with bell-shaped velocity profiles in both subjects with PD and healthy controls.

**Figure 3:**
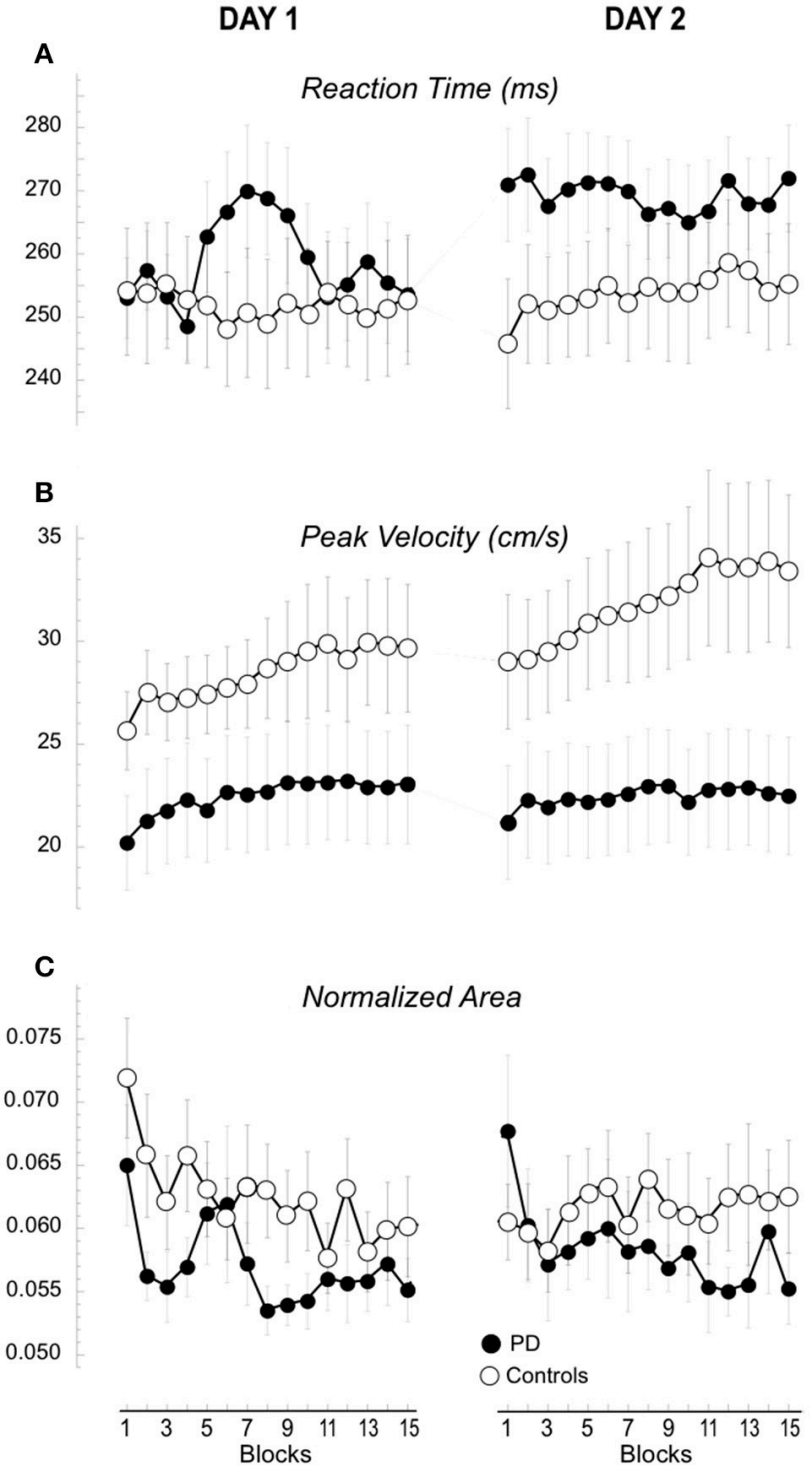
**Performance measures of the 15 blocks in Day 1 and 2**. Means (±SE) of the changes of reaction time **(A)**, peak velocity **(B)**, and hand path area **(C)** for each block are plotted separately for day 1 and 2 and for the two groups, the controls (empty circles) and the patients with PD (filled circles).

The results of mixed model ANOVA comparing the performance indices of the two groups across the 15 blocks in the 2 days are reported in Table 1 and illustrated in Figure 3. Briefly, analysis of reaction times (Figure 3A) did not reveal significant effects of group, blocks or days. However, significant interactions (see Table 1) prompted further analyses that, in controls, showed that reaction times were stable across days and blocks [mixed model ANOVA, day: *F*_(1, 10)_ = 0.293, *p* = 0.598; blocks: *F*_(14, 168)_ = 0.510, *p* = 0.925; day X blocks: *F*_(1, 14)_ = 1.61, *p* = 0.181]. Conversely, in the patient group, reaction times increased in the second compared to the first day but not for all blocks [day: *F*_(1, 10)_ = 7.09, *p* = 0.024; blocks: *F*_(14, 140)_ = 2.24, *p* = 0.08; day X blocks: *F*_(1, 14)_ = 2.25, *p* = 0.05].

**Table 1:** **Results of mixed model ANOVA comparing performance indices and modulation depth of the two groups across blocks and days**.

	**Group**		**Day**		**Block**		**Group x day**		**Group x block**		**Block x day**		**Group x day x block**	
	***F*_(1, 22)_**	**P**	***F*_(1, 22)_**	***P***	***F*_(14, 616)_**	***p***	***F***	***p***	***F***	***p***	***F***	***p***	***F***	***p***
Reaction Time	0.82	0.376	2.76	0.111	1.15	0.318	5.64	**0.027**	1.99	**0.019**	3.05	**0.000**	1.22	0.259
Peak Velocity	3.53	0.074	6.81	**0.016**	6.66	**0.000**	6.39	**0.019**	1.80	**0.038**	0.32	0.991	0.88	0.586
Normalized Area	2.76	0.111	0.05	0.835	3.43	**0.000**	0.49	0.492	0.92	0.539	1.27	0.223	2.43	**0.003**
Modulation Depth	2.52	0.127	0.38	0.542	7.97	0.000	0.65	0.827	3.29	**0.000**	0.53	0.914	1.83	**0.034**

*Bold values indicate significance p < 0.05*.

The amplitude of peak velocities was highly correlated with the amplitude of peak acceleration and movement times (*r*^2^ > 0.8), (Perfetti et al., 2011; Moisello et al., 2015b). Therefore, here we focus on the changes in peak velocity. The amplitude of peak velocity (Figure 3B, Table 1) was in general lower in patients compared to controls and significantly increased across blocks and days. A significant interaction Group X Day suggested a different between-day increase for the two groups. Separate group analyses revealed that, in controls, peak velocity significantly and constantly increased across blocks and days [day: *F*_(1, 12)_ = 8.42, *p* = 0.013; blocks: *F*_(14, 168)_ = 5.05, *p* = 0.027 c; day X blocks: *F*_(1, 14)_ = 0.47, *p* = 0.950]. Interestingly, the average peak velocity of the last block of Day 1 was similar to the average of the first block of Day 2 (Figure 3B, 29.6 ± 11.18 cm/s; 29.1 ± 11.78 cm/s, paired *T*-test: *p* = 0.21), suggesting a good retention in the controls. In patients, peak velocity increased only across blocks but not across days [day: *F*_(1, 10)_ = 0.02, *p* = 0.890; blocks: *F*_(14, 140)_ = 3.46, *p* = 0.035 c; day X blocks: *F*_(1, 14)_ = 1.19, *p* = 0.289]. Further inspection of the data revealed that the average peak velocity of the first block of Day 2 was lower than the average of the last block of Day 1 (23.04 ± 9.53 cm/s; 22.09 ± 9.13 cm/s, paired *T*-test: *p* = 0.033, Figure 3B), suggesting a poor retention for this kinematic measure. Similar results were obtained for normalized area, a measure of spatial accuracy and inter-joint coordination (Figure 3C, Table 1). This measure decreased significantly across blocks with a significant Block X Day X Group. Separate analyses showed that in the controls values decreased across blocks on day 1 and these values were maintained on day 2 testing [day: *F*_(1)_ = 0.093, *p* = 0.765; blocks: *F*_(4.91)_ = 1.125, *p* = 0.357 c; day X blocks: *F*_(5.367)_ = 3.197, *p* = 0.01]; importantly, the average of the last block of Day 1 (0.0605 ± 0.0108) was similar to the average of the first block of day 2 (0.0602 ± 0.0142; 0.0605 ± 0.0108; paired *t*-test *p* = 0.43, see Figure 3C). Conversely, in the patient group normalized area indices were similar in both days with a similar decrease across blocks [Figure 3C, day: *F*_(1)_ = 0.721, *p* = 0.416; blocks: *F*_(2.5)_ = 2.5, *p* = 0.08 c; day X blocks: *F*_(4.235)_ = 0.688, *p* = 0.612] and the values of the first block on day 2 was significantly greater than those of the last block of day 1 (0.0677 ± 0.0227; 0.0551 ± 0.0083; paired *t*-test: *p* = 0.024; Figure 3C).

In summary, performance improvement occurred in both groups in terms of increased peak velocity and decreased normalized area across blocks. However, while normal subjects retained most of these improvements the following day, in the patient group retention was significantly reduced.

### Beta modulation depth does not change across days

**Figure 4:**
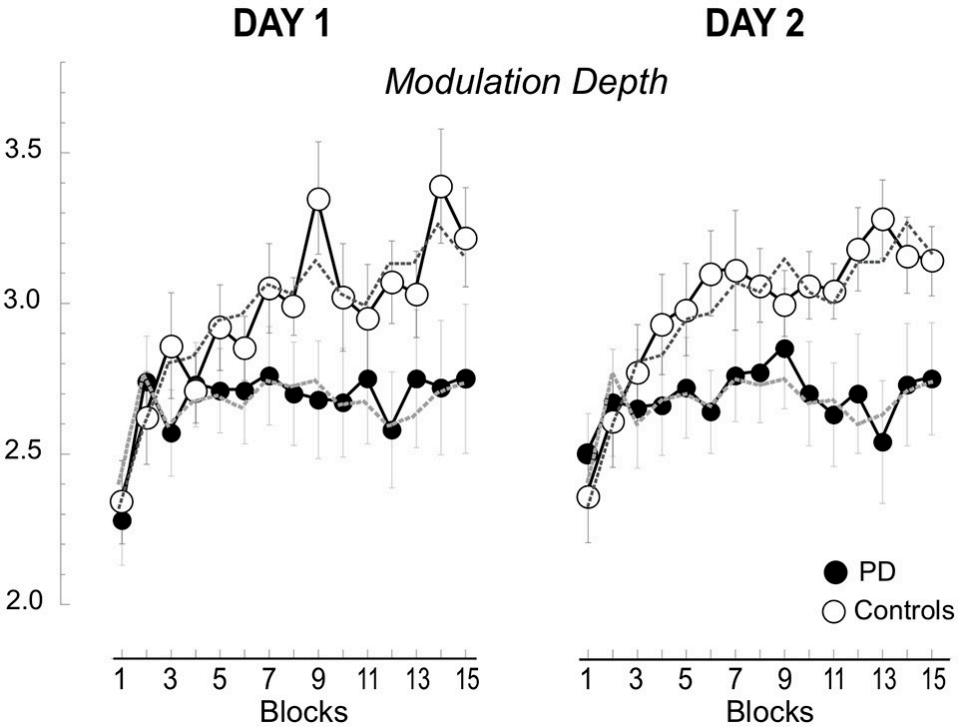
**Beta modulation depth of the 15 blocks in Day 1 and 2**. Means (±SE) of the changes of modulation depth for each block are plotted separately for day 1 and 2 and for the two groups, the controls (empty circles) and the patients with PD (filled circles). The values for the 2 days are basically overlapping. The dotted lines represent the averages across the 2 days.

As described in the methods, we measured the modulation depth of beta power by computing the difference between the ERD and ERS peaks. Then, we verified the differences between the two groups on day 1 and 2 across the 15 blocks with a mixed model ANOVA. The results are reported in Table 1 and illustrated in Figure 4. As in our previous report (Moisello et al., 2015b), we found that modulation depth increased across blocks mostly in the controls (Table 1). The new finding is that the same across-block increase was present the following day starting and ending within the same value range of the previous day (Figure 4). Separate group analyses confirmed that in controls, there was a significant across-block increase of modulation depth that was similar in the 2 days [block: *F*_(14, 168)_ = 11.1, *p* < 0.0001; day: *F*_(1, 12)_ = 0.176, *p* = 0.682; block X day: *F*_(14, 1)_ = 1.71, *p* = 0.159]. On the other hand, in the patient group, modulation depth was similar in the 2 days and increased slightly, but not significantly, across the blocks [Block: *F*_(14, 140)_ = 1.223, *p* = 0.265; Day: *F*_(1, 10)_ = 0.257, *p* = 0.623; Block X Day: *F*_(14, 1)_ = 0.713, *p* = 0.759].

Interestingly, in the control group the increase of modulation depth from block 1 to 15 on Day 1 was significantly correlated with decrease of hand path area across days [that is: (Day 2 Block 1-Day 1 Block 15)/Day 1 Block 15 in percentage, see Supplemental Material Figure 1A; *r* = 0.74; *p* = 0.004]. No significant correlations were found for the patient group.

Finally, as in our previous study, (Moisello et al., 2015b), the change of modulation depth across blocks mainly reflected an increase in beta ERS amplitude, as illustrated in Supplemental Material Figure 2.

In summary, modulation depth significantly increased across the blocks only in the control group, while in the patient group such an increase was minimal and did not achieve significance. The following day, modulation depth returned to baseline values in both groups to increase again across blocks, without significant differences compared to the previous day. Finally, in the controls, retention of kinematic skill, in terms of hand path area, was linked to the increases of modulation during Day 1, so that the higher the increase in modulation, the greater the retention.

### Modulation depth shows increases within blocks in both groups but between-block savings only in the controls

To determine whether the progressive increase of modulation depth across block developed within each block, we compared the values of the first 16 (*early*) and the last 16 (*late*) trials of each block. Based on the finding that the power dynamic was basically identical in the 2 days, in order to increase the signal-to-noise ratio and the number of trials, we merged the data of day 1 and 2. We first focused our analyses on the controls, as only in this group we found a significant increase of modulation depth across blocks.

#### Control group

**Figure 5:**
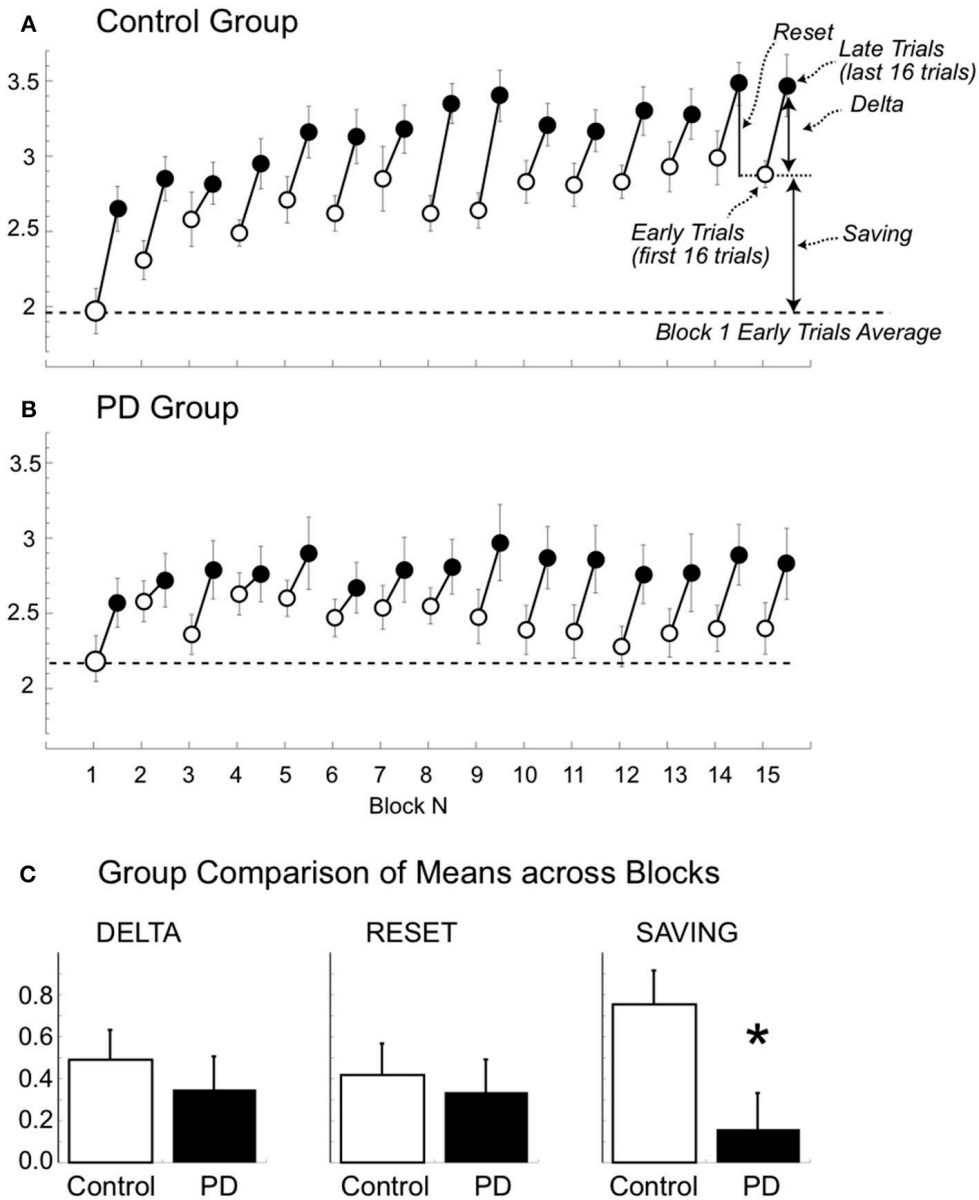
**Beta modulation depth of the early and late trials for the 15 blocks (A,B)**. Mean (±SE) beta modulation depth of the controls **(A)** and patient group **(B)** are plotted for the early (first 16 trials, empty circle) and late (last 16 trials, filled circle) trials for each of the 15 blocks. Data of day 1 and 2 were combined. The horizontal dotted and continuous lines represent the minimum and maximum mean values, respectively. In **(A)** we also illustrate the delta (i.e., difference between the modulation of early and late trials), the reset (i.e., difference in modulation between late trials of one block and the early trials of the following one), and the saving (i.e., modulation difference of the early trial of the first block and the early trial of the other blocks). **(C)** Comparison of mean (±SE) delta, reset and saving in the two groups. Notice that the only significant difference was found for mean savings (asterisk).

The results of the repeated measures ANOVA (trials and blocks) revealed that modulation depth was greater in the late compared to the early trials and that modulation depth of both early and late trials generally increased with the block number [Figure 5A, trial: *F*_(1, 12)_ = 58.9, *p* < 0.0001; block: *F*_(14, 168)_ = 8.130, *p* < 0.0001; trial X block: *F*_(1, 14)_ = 1.268, *p* = 0.291]. The lack of trial and block interaction suggests that the modulation increase within each block (difference between the early and late trials in each block or delta, see Figure 5A) had similar magnitude across blocks, as also confirmed by the result of an ANOVA directly testing the effect of blocks on the delta values [*F*_(13, 168)_ = 0.96, *p* = 0.50].

Interestingly, the increase of beta modulation carried from the end of a block on to the beginning of the next one was only partial, indicating that a *between-block reset* (see Figure 5A) occurred. In fact, on average, modulation of *late* trials of a given block was greater than that of the *early* trials of the following block [3.14 ± 0.61 vs. 2.72 ± 0.55; repeated measure ANOVA: trial: *F*_(1, 156)_ = 40.133, *p* < 0.0001; block: *F*_(13, 156)_ = 6.627, *p* < 0.0001; trial X block: *F*_(1, 13)_ = 1.094, *p* = 0.373, see Figure 5A]. A factor contributing to the *between-block reset* could have been the time interval between the end of a block and the beginning of the following one. Indeed, as noted in the methods, the time intervals in controls varied from 13 to 249 s (mean ±SD: 29.8 ± 31.6 s). While we did not find any significant linear correlations between time intervals and *between-block* reset in either block- or subject-based analyses, blocks with time interval shorter than 90 s had, on average, smaller values of *between-block reset*s (*N* = 342, mean ±SD: 0.40 ± 0.74) compared to those with longer time intervals (*N* = 22, mean ±SD: 0.72 ± 0.56, *p* = 0.03). Therefore, it is possible that only very long periods of inactivity (more than 90 s) might contribute to the between-block reset. In any event, the reset never pushed the modulation depth of the *early trials* to the values of the *early trials* of the first block. In fact, the modulation of the early trials of block 1 was always significantly lower than that of the early trials of all the other blocks (all paired *t*-test: *p* < 0.0036).

Finally, we found no significant trial-related changes in kinematic variables and no significant correlations between such changes and the trial-related changes of beta power.

#### Patients with PD

Similarly to the controls, the values of modulation depth in the patient group (Figure 5B) were greater for the late compared to early trials [trial: *F*_(1, 10)_ = 11.828, *p* = 0.006]. Such differences were more evident and statistically significant starting from block 8 (two-tailed paired *t*-tests, late vs. early trials: *p* < 0.05, see Figure 4). Nevertheless, differently from the controls, modulation depth did not change significantly across blocks for both early and late trials [block: *F*_(14, 140)_ = 0.883, *p* = 0.479; trial X block: *F*_(1, 14)_ = 1.094; *p* = 0.375]. In other words, in the patient group, the trend of modulation depth was to increase during each block like in the controls. However, compared to the controls, the patients' modulation depth of both early and late trials reverted closer to block 1′s values in each subsequent block, with almost complete between-block reset and without substantial savings. It is unlikely that the lack of between-blocks savings in PD was due to increased time interval between blocks, as the time intervals in PD (mean ±SD: 29.95 ± 30.32 s; range: 13–249 s) were not significantly different from those of controls (two-tailed unpaired *t*-test: *p* = 0.95).

As in the control group, analyses in the PD group's data did not reveal either significant trial-related changes in kinematic variables or significant correlations between those changes and the trial-related changes of beta power, disease characteristics and medication levels.

#### Group comparisons

The results of mixed model ANOVAs with direct comparisons between the two groups are reported in Table 2 and illustrated in Figure 5C. In summary, modulation depth of the early trials was greater in controls than in patients starting from block 10 (*post-hoc t*-tests: all *p* < 0.03), with significant greater savings (i.e., differences between early trials of block 1 and those of all the other blocks) starting from block 4 (*post-hoc t*-tests: all *p* < 0.03). Marginal, non-significant differences between the two groups were found for modulation depth of the late trials, while no differences were found for the values of delta and reset. Interestingly, analyses of the two groups, singly and in combination, revealed that resets were highly correlated with delta values, in that the greater the deltas the higher the resets values (controls: *r* = 0.97; *p* < 0.0001; PD: *r* = 0.99; *p* < 0.0001; all: *r* = 0.98; *p* < 0.0001, Supplemental Material Figure 3), but not with savings (controls: *r* = 0.04; *p* = ns; PD: *r* = 0.42; *p* = ns; all: *r* = 0.27; *p* = ns).

**Table 2:** **Results of mixed model ANOVA comparing modulation depth of early and late trials as well as of delta, reset and savings of the two groups across blocks**.

	**Group**		**Block**		**Group x block**	
	***F*_(1, 22)_**	***p***	***F*_(14, 308)_**	***p***	***F***	***P***
Early trials modulation	3.761	**0.000**	3.761	**0.000**	4.337	**0.000**
Late trails modulation	2.930	0.098	3.860	**0.000**	1.372	0.165
Modulation delta	1.490	0.0235	1.000	0.453	1.318	0.195
Modulation reset	0.0538	0.471	1.954	**0.025**	0.841	0.616
Modulation savings	9.013	**0.007**	112.900	**0.000**	6.698	**0.000**

*Bold values indicate significance p < 0.05*.

Altogether, these results suggest that in PD, as opposed to normal subjects, the increase in beta power reached at the end of each block was not carried on in a significant extent to each successive block, thus contributing to the lack of significant increase of mean modulation depth across blocks.

### Resting state beta power locally increases in controls but not in patients in both days

We first explored the practice-related changes of beta power at rest in the two sessions of the control group (Supplemental Material Figure 1C). Briefly, we found that resting beta power increased significantly after practice without a significant difference between the two sessions [Supplemental Material Figure 1C; practice: *F*_(1, 12)_ = 8.5, *p* = 0.01; day: *F*_(1, 12)_ = 4.1, *p* = 0.07; practice X day: *F*_(1, 1)_ = 0.01, *p* = 0.92]. As expected, *post-hoc* tests also revealed that PRE2 values did not significantly differ from PRE1 (one-tailed *T*-test: *p* > 0.1) but were significantly lower than POST1 (one-tailed *T*-test: *p* = 0.004) suggesting that the significant increase of beta power after practice on day 1 (one-tailed *T*-test: *p* = 0.01) was not present anymore the following day before the start of the second session. Interestingly, the changes in resting state on day 1 were mildly, but significantly correlated with the retention of the improvement in hand path area (*r* = 0.62, *p* < 0.05; Supplemental Material Figure 1B), suggesting that the increase in resting state partially reflects processes that are linked to skill retention. Interestingly, changes in resting state also showed significant correlations with delta and reset values (*r* = 0.66, *p* < 0.01; *r* = 0.72, *p* < 0.005, respectively), in that the greater the increase of beta at rest after practice the greater the delta and reset during the practice blocks.

In the patient group, we found a slight increase of beta power that did not reach significance in either session [Supplemental Material Figure 1D; practice: *F*_(1, 10)_ = 0.92, *p* = 0.36; day: *F*_(1, 10)_ = 0.88, *p* = 0.37; practice X day: *F*_(1, 1)_ = 0.10, *p* = 0.76]. We did not find any significant correlation with resting state changes in the patient group.

## Discussion

The results of this paper confirm our previous findings that the depth of movement-related beta modulation increases across performance blocks in normal subjects but not in patients with PD, while performance improves in both groups (Moisello et al., 2015b). In addition, we have two sets of novel findings. The first is that beta modulation depth increases with practice within the same range when repeated the following day. Importantly, the controls retained most of kinematic improvements achieved at the end of day 1; such retention was proportional to the increase in beta modulation of the first session: the higher the increase in modulation on the first session, the better the performance at the beginning of the second session. Conversely, the patient group did not show significant retention on day 2. The other novel finding is that, in controls, beta modulation increased within each block and the increase reached at the end of each block was largely carried on to the successive block. The amount of the carry-overs was not related to changes of behavioral indices. In the patient group, no significant between-block carry over was found, thus contributing to the lack of significant increase of mean modulation depth across blocks and possibly to abnormal beta ERS-ERD reported in other papers (Degardin et al., 2009; Heinrichs-Graham et al., 2014; Canessa et al., 2017). Altogether, these results suggest that movement-related beta modulation changes during practice could reflect two phenomena that will be discussed in details in the following paragraphs. Briefly, on one side, the intra-block increase might represent the growing reliance on feed-forward mechanisms that facilitate performance during that same session. On the other hand, one might speculate that the global increase from the first to the last block could underlie the biochemical mechanisms that induce retention of a motor skill. This interpretation can explain why the performance of patients improves on line, during the task, but do not show retention of the improvement the day after.

### Improvement and retention of hand-path area, peak velocity and beta modulation

One of the novel and most important findings is that, in the control group, the improvements of both hand path area and peak velocity reached at the end of day 1 were maintained during the testing of the second day. This was not the case of the patients where retention for both measures was lower. On-line increases in the amplitude of peak velocities–which paralleled decreases in movement times as reported in the previous paper (Moisello et al., 2015b) and the decreases in hand path areas were present in both groups and in both days. However, we found that only the retention indices of hand path area and not those of peak velocity correlated with the increases in beta modulation that occurred in day 1. The amplitudes of peak velocity and acceleration, i.e., the first and second-time derivatives of movement extent, respectively, reflect motor planning, advanced preparation processes or feedforward mechanisms. In other words, the progressive increase in peak velocity and acceleration represents an increasing reliance on feedforward mechanisms (Ghez and Gordon, 1987; Gordon et al., 1994). The absence of significant correlations between the changes of beta modulation and peak velocities can be explained by previous evidence that planning of peak velocities and acceleration is more dependent on oscillations in the low alpha range over the parietal regions (Perfetti et al., 2011).

Hand path area is an index of trajectory accuracy that reflects inter-joint coordination (Sainburg et al., 1995; Huber et al., 2006; Moisello et al., 2008). Proper inter-joint coordination is needed to overcome the interaction torques that develop during the movement. Importantly, movement speed affects inter-joint coordination: the faster the movement the greater the errors induced by interaction torques (Sainburg et al., 1995). This prompts three considerations. First, the improvement of hand path area occurred despite the steady increase in peak velocity in the controls and patients alike. Second, the lower peak velocities of the patients with PD can explain their lower (and thus better) values of hand path areas compared to normal subjects. Third, the considerable increase of peak velocities of day 2 compared to day 1 in the controls but not in the patients makes the retention in terms of hand path area in the control group even more notable. Inter-joint coordination, and thus hand path area, relies on efficient processing of proprioceptive information (i.e., feedback type of mechanisms) and accuracy of internal models or sensory-motor memories (i.e., feedforward type of mechanisms) (Sainburg et al., 1995; Huber et al., 2006; Moisello et al., 2008). Studies in patients without proprioception (Ghez et al., 1995; Gordon et al., 1995; Sainburg et al., 1995) have revealed large increases of hand path area due to the uncoupling of joint motions that resulted from deficits of agonist and antagonist muscles action in compensating for the interaction torques (Sainburg et al., 1995). Indeed, on-line proprioceptive information is fundamental to adjust the muscles' activation pattern to overcome the interaction torques that develop between limb segments during their motion (Sainburg et al., 1995). However, proper processing of on-line information by itself is not sufficient to correct entirely for interaction torques overall when movement is fast and hand position is changing rapidly, as corrective responses will occur too late due to delays in neural transmission and muscle contraction. This is where internal models or memories of limb dynamics must intervene to perfect intersegmental dynamics: feedforward mechanisms are used to form and update such memories and thus to program movements in advance taking into account limb dynamics. Unquestionably, in normal subjects, hand path area decreases when a reaching task is repeated hours or days after the original training (Moisello et al., 2008), thus suggesting that sensory-motor memory and its updating and maintenance are involved in counteracting interaction torques. This is also in agreement with the results of our studies showing that temporary limb immobilization produces increases of both inter-joint timing and hand path area. Such changes correlated with electrophysiological alterations of proprioceptive information processing within the cortical sensory-motor areas (Huber et al., 2006; Moisello et al., 2008). The present findings in controls also suggest that retention of improvement in this index is linked to the increase of beta modulation from the beginning and the end of the first training session both during the task and at rest, thus further linking beta power to proprioceptive information and memory processes in the sensory-motor areas. Retention was not significantly present in patients with PD. Both groups, however, displayed on line improvements both for peak velocity and hand path areas accompanied by similar within-block increases in beta movement-related modulation. It is thus possible that such beta changes reflect a major shift of the performance toward feedforward mechanisms. Although further experiments are needed to support this conclusion, this view is in line with recent studies on visuo-motor adaptation suggesting that changes in beta modulation indices reflect cortical sensory processing and the updating of internal models (Tan et al., 2016).

### Between-block carry-overs are significant only in the control group

The second novel finding is that movement-related beta modulation increased within each block from the early to the late trials and the increase reached in the late trials persisted in the early trials of the successive block, to a significant extent in the controls and much less so in the patient group. The poor between-block carry over in the patient group explained the fact that beta movement-related modulation depth did not increase significantly across the 40-min sessions. However, even in normal subjects, the increase carried from the end to the beginning of successive blocks was only partial. The amount of this *between-block reset* was not linearly dependent on the interval between blocks, although time intervals >90 s corresponded to higher values of *between-block reset*s. Therefore, it is possible that only very long periods of inactivity might contribute to the between-block reset, as at the beginning of the second session 24 h later. In any event, in normal subjects the *between-block* reset never pushed beta movement-related modulation depth of the early trials of successive blocks to the values of the early trials of the first block, while in patients, despite the time interval between blocks was basically overlapping to that of controls, there were no significant savings or carryovers.

What could be the cause or causes of such resettings and savings? And why savings are decreased in PD? While a clear explanation is not readily available, it is unlikely that resettings and savings are related to changes in performance attributes, as there were no correlations with any of the performance indices. As beta movement-related modulation likely reflects the interplay of sensory and motor regions' activities (Shimazu et al., 1999; Cassim et al., 2000), the continuous performance in a motor task such as ours might provide the basis for user-dependent plasticity induction. The biochemical and electrophysiological mechanisms of LTP have been characterized in slices and cell preparations. Briefly, while the late phases of LTP depend on gene transcription and protein synthesis thus requiring several hours or days, its earliest phases are independent of protein synthesis and usually last for a few minutes or hours. These phases are characterized by the phosphorylation of existing AMPA receptors and the insertion of other AMPA receptors so that upcoming excitatory stimuli can produce larger postsynaptic responses. These phenomena are usually short lasting and might decay within minutes in absence of a continuous stimulation. Experimental studies have demonstrated that PD is associated with alterations in the AMPA receptor subunit composition and NMDA/AMPA receptor ratio, thus preventing an efficient induction of the early phases of LTP (Paillé et al., 2010). Therefore, if the increase of movement-related beta modulation during the task parallels the induction of LTP and is independent of on-line performance improvement, one should expect increases of beta modulation within a set of consecutive movements and a partial decrease between successive movement sets with a decrease in skill retention as we found in PD. Of course, these conclusions remain for the moment speculations.

Finally, we need to consider that our patients with PD were tested during optimal pharmacological condition. As previously reported (Degardin et al., 2009; Canessa et al., 2017) and as seen also for behavioral data, dopaminergic therapy might partially, but not completely, compensate for ERS/ERD abnormalities and, in our case, possibly produce normal delta values. However, the aim of this study was to characterize movement-related beta oscillations changes with extended practice: it would have been hard for patients to work for 40 min without stopping or experiencing great fatigue and it would have been difficult to standardize drugs withdrawal over 2 days since many participants were taking long-acting drugs, such as dopamine-agonists. Further studies are indeed to define the effect of dopaminergic drugs on the described phenomena and its abnormalities.

In summary, while the intra-block increase of movement-related modulation might represent a reinforcement of feed-forward mechanisms that facilitate performance, the total beta power increase from the first to the last block could be related to the mechanisms that induce retention of a motor skill and could represent a sort of *capacity* for use-dependent plasticity within the sensory-motor system rather than the explicit coding of specific movement characteristics.

## Ethics statement

This study was carried out in accordance with the recommendations of the IRBs of CUNY and NYU with written informed consent from all subjects. All subjects gave written informed consent in accordance with the Declaration of Helsinki. The protocol was approved by the IRBs of CUNY and NYU.

## Author contributions

Study conception: MFG, CC, GT, GF, and AD. Study design: MFG, II, AQ, CM, and AN. Acquisition: AD, PP, JL, and CM. Analyses: MFG, AC, SR, PP, JL, CM, and AN. Interpretation of the results: MFG, II, AQ, AC, and AN. Manuscript drafting: MFG, CC, AQ, AC, PP, JL, CM, and AN. Manuscript revising: MFG, CC, GT, II, GF, AQ, AD, AC, SR, and AN. All the authors agreed to be accountable for all aspects of the work in ensuring that questions related to the accuracy or integrity of any part of the work are appropriately investigated and resolved.

### Conflict of interest statement

The authors declare that the research was conducted in the absence of any commercial or financial relationships that could be construed as a potential conflict of interest.
