# Supplementary figures and images for: Beta Oscillatory Changes and Retention of Motor Skills during Practice in Healthy Subjects and in Patients with Parkinson's Disease
Source: Front Hum Neurosci. 2017 Mar 7;11:104. doi: 10.3389/fnhum.2017.00104 (PMC5339296; doi:10.3389/fnhum.2017.00104)

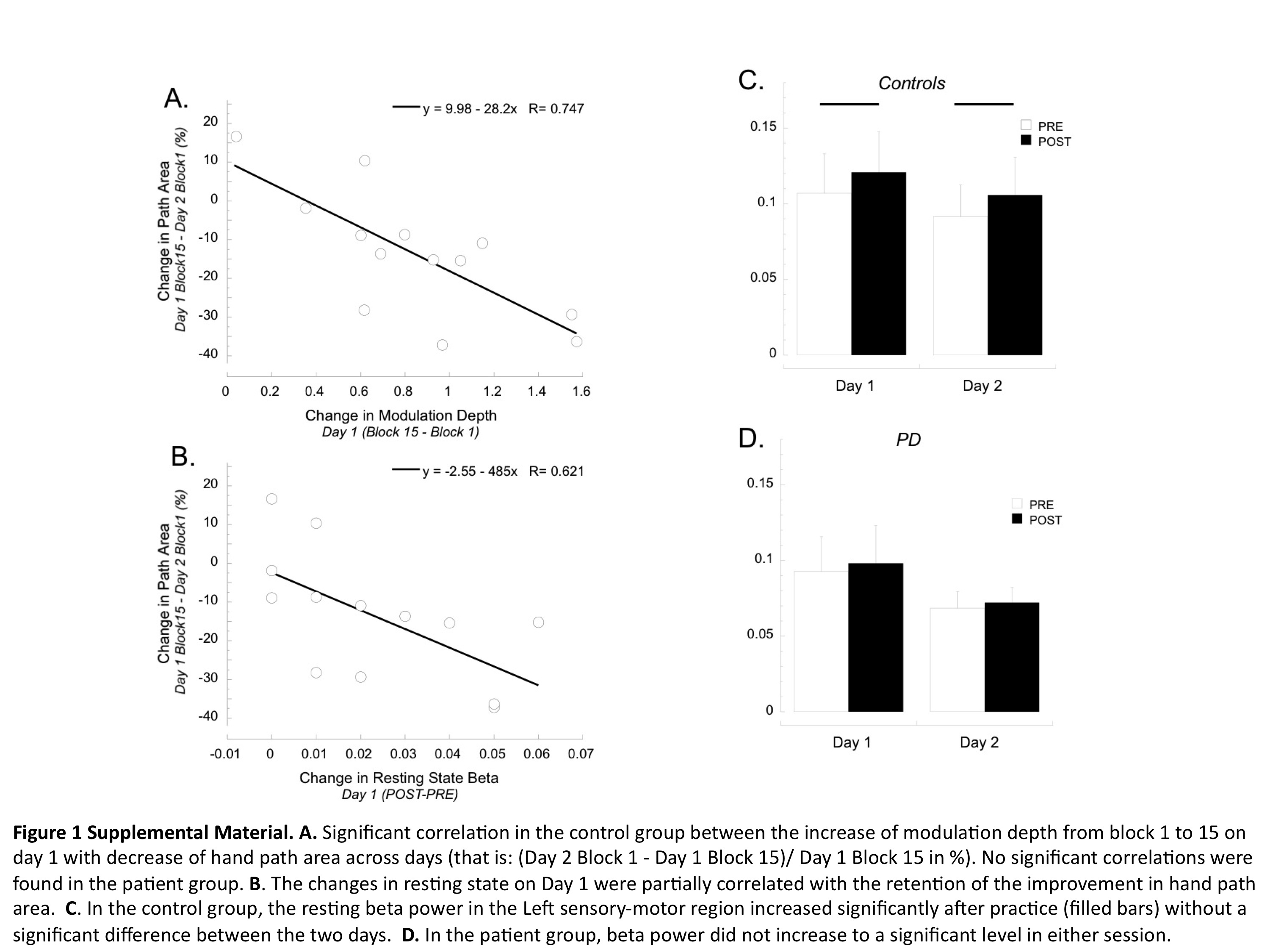

Supplement: Supplementary file 1 [file Image1.jpg]

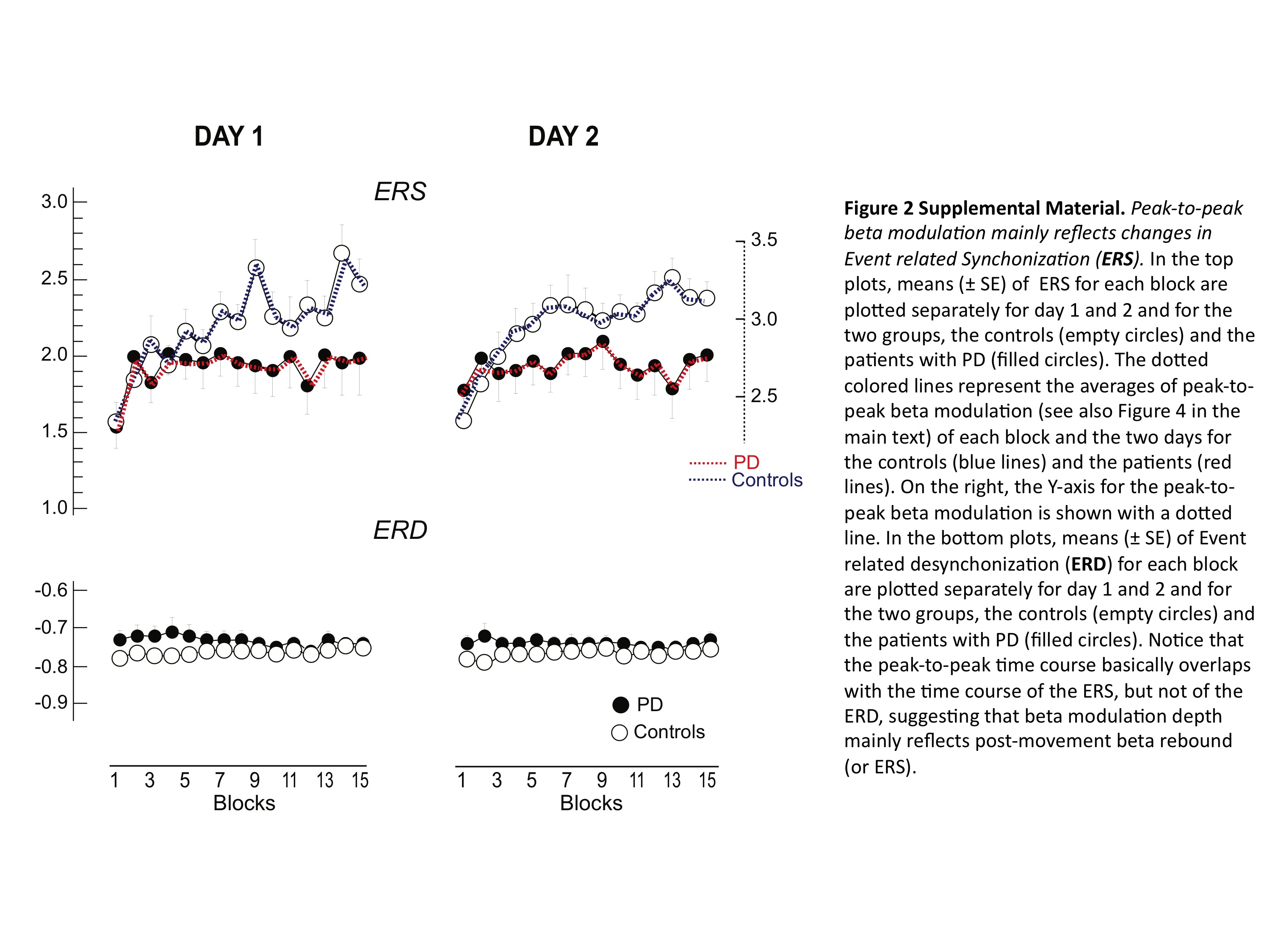

Supplement: Supplementary file 2 [file Image2.jpg]

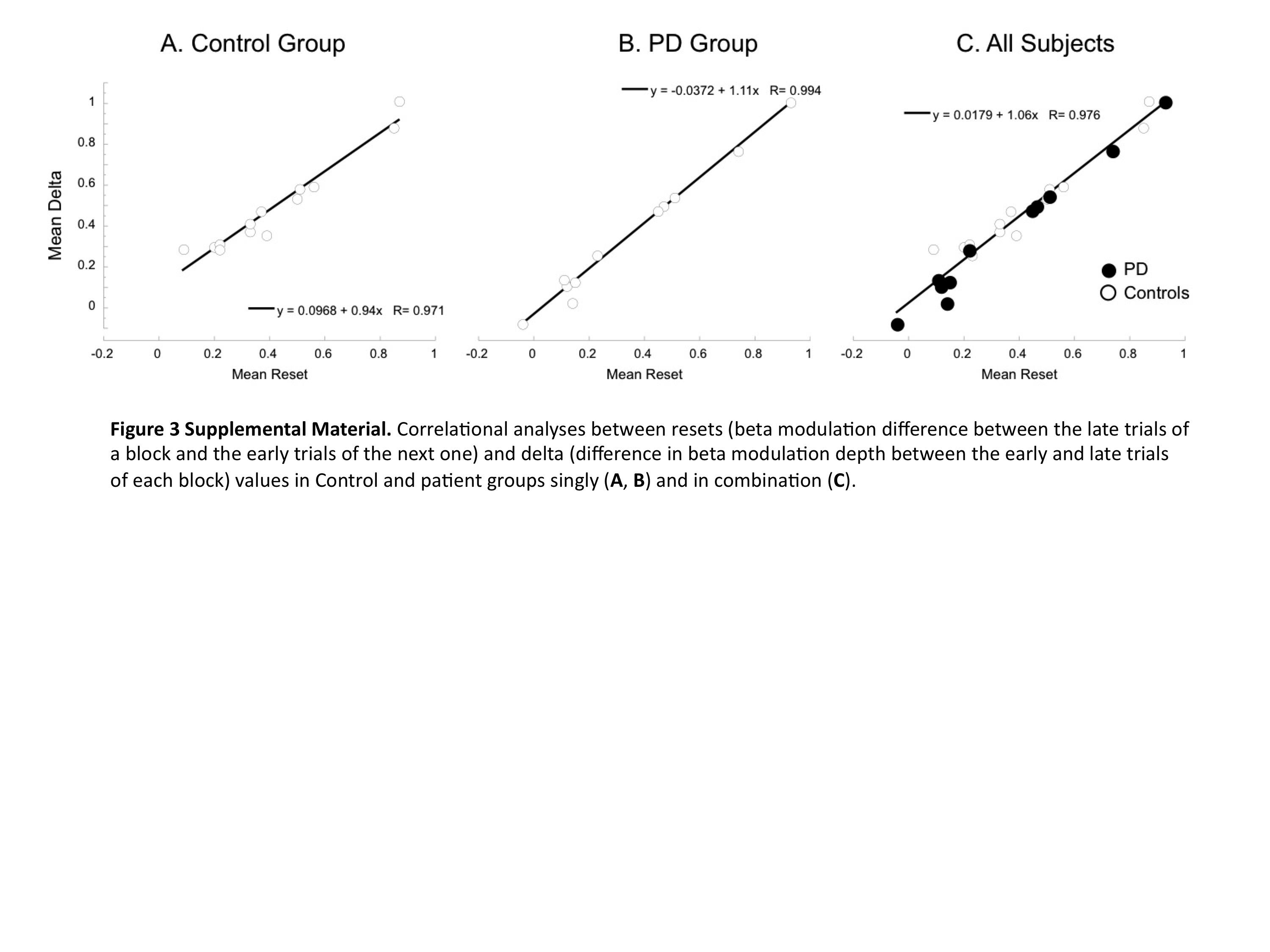

Supplement: Supplementary file 3 [file Image3.jpg]
